# Incorporating temporal dynamics of mutations to enhance the prediction capability of antiretroviral therapy’s outcome for HIV-1

**DOI:** 10.1093/bioinformatics/btae327

**Published:** 2024-05-22

**Authors:** Giulia Di Teodoro, Martin Pirkl, Francesca Incardona, Ilaria Vicenti, Anders Sönnerborg, Rolf Kaiser, Laura Palagi, Maurizio Zazzi, Thomas Lengauer

**Affiliations:** Department of Computer Control and Management Engineering Antonio Ruberti, Sapienza University of Rome, Rome 00185, Italy; EuResist Network, Rome 00152, Italy; Institute of Virology, Faculty of Medicine and University Hospital Cologne, University of Cologne, Cologne 50935, Germany; German Center for Infection Research (DZIF), Cologne 50935, Germany; EuResist Network, Rome 00152, Italy; Department of Medical Biotechnologies, University of Siena, Siena 53100, Italy; I-PRO, Rome 00152, Italy; Department of Medicine Huddinge, Karolinska Institutet, Division of Infectious Diseases, Stockholm 14152, Sweden; Department of Infectious Diseases, Karolinska University Hospital, Stockholm 14186, Sweden; Institute of Virology, Faculty of Medicine and University Hospital Cologne, University of Cologne, Cologne 50935, Germany; German Center for Infection Research (DZIF), Cologne 50935, Germany; Department of Computer Control and Management Engineering Antonio Ruberti, Sapienza University of Rome, Rome 00185, Italy; I-PRO, Rome 00152, Italy; Institute of Virology, Faculty of Medicine and University Hospital Cologne, University of Cologne, Cologne 50935, Germany; Computational Biology, Max Planck Institute for Informatics, Saarbrücken 66123, Germany

## Abstract

**Motivation:**

In predicting HIV therapy outcomes, a critical clinical question is whether using historical information can enhance predictive capabilities compared with current or latest available data analysis. This study analyses whether historical knowledge, which includes viral mutations detected in all genotypic tests before therapy, their temporal occurrence, and concomitant viral load measurements, can bring improvements. We introduce a method to weigh mutations, considering the previously enumerated factors and the reference mutation-drug Stanford resistance tables. We compare a model encompassing history (H) with one not using this information (NH).

**Results:**

The H-model demonstrates superior discriminative ability, with a higher ROC-AUC score (76.34%) than the NH-model (74.98%). Wilcoxon test results confirm significant improvement of predictive accuracy for treatment outcomes through incorporating historical information. The increased performance of the H-model might be attributed to its consideration of latent HIV reservoirs, probably obtained when leveraging historical information. The findings emphasize the importance of temporal dynamics in acquiring mutations. However, our result also shows that prediction accuracy remains relatively high even when no historical information is available.

**Availability and implementation:**

This analysis was conducted using the Euresist Integrated DataBase (EIDB). For further validation, we encourage reproducing this study with the latest release of the EIDB, which can be accessed upon request through the Euresist Network.

## 1 Introduction

Human immunodeficiency virus (HIV), if untreated, is a deadly pathogen. While there are treatment options, there is still no cure or vaccine. Since its discovery in 1981, 84.2 [64.0–113.0] million people have been infected with HIV, claiming about 40.1 [33.6–48.6] million lives. By the end of 2022, about 39.0 million people were living with HIV (World Health Organization 2023). Early diagnosis and proper treatment offer life expectancy comparable to HIV-negative individuals. HIV-1 infection requires antiretroviral treatment; without it, patients eventually develop acquired immunodeficiency syndrome (AIDS). Standard care involves the administration of cocktails of antiretroviral drugs, rather than one individual medicine, to minimize the risk of emergent drug resistance. Antiretroviral therapies against HIV-1 can lead to the selection of drug-resistant HIV-1 variants that can spread between hosts (Van De Klundert *et al.* 2022), causing treatment failure (Langford *et al.* 2007, Wensing *et al.* 2022). Resistance testing can help to suppress viral replication and to prevent the transmission of resistant variants. The analysis of the susceptibility of the HIV-1 variants to available antiretroviral drugs can be facilitated via genotypic testing or phenotypic testing (Tang and Shafer 2012). Phenotypic testing directly measures the drug concentration required to inhibit virus replication *in vitro*, whereas genotypic testing comprises sequencing the viral genome and inferring drug susceptibility based on prior knowledge of resistance mutations. Genotypic resistance tests, being the more practical variant, are routinely used in clinical practice. If drug-resistant viral variants emerge, therapy must be replaced with a different drug combination to suppress replication. Clinicians struggle with identifying new drug combinations to suppress HIV replication, taking into account viral drug resistance, previous successful and failing therapies, and retention of future treatment options. However, the latent viral population in the patient living with HIV (PLWH) accumulates a large number of resistance-associated mutations, which may not be observable in blood serum but rapidly reappear when it is advantageous, i.e. under the appropriate drug pressure (Rhee *et al.* 2022, Wensing *et al.* 2022). The many possible drug combinations complicate therapy selection, especially in advanced stages. For this reason, over the years genotypic interpretation systems for drug resistance have been developed for predicting the success or failure of antiretroviral drug regimens.

Genotypic drug resistance interpretation systems (GIS) are rules-based or data-driven interpretation systems. The former approach uses tables of drug-resistance mutations assembled by expert groups (Wensing *et al.* 2022) to calculate drug-resistance scores that assess drug resistance of viral genotypes. Several rules sets have been developed over the years, such as the ones from ANRS, HIVdb (Tang *et al.* 2012), HIV-GRADE, and the Rega Institute, all available on the HIV-GRADE website (Obermeier *et al.* 2012). These rules-based systems are continually updated according to changes in observed HIV drug resistance, treatment guidelines, and expert opinion (Paredes *et al.* 2017). All of these systems interpret data obtained from Sanger sequencing, a long-standing technology widely used thanks to its low rate of error and its cost-effectiveness. However, this technique has limits in detecting minor resistant populations (Tsiatis *et al.* 2010, Davidson *et al.* 2012) that could have clinical relevance (Cozzi-Lepri *et al.* 2015, Vrancken *et al.* 2016). High-throughput sequencing techniques, collectively referred to as next-generation sequencing (NGS) (Fox *et al.* 2014), are now being increasingly used which can detect minority variants representing as low as 1% of the viral population, as opposed to roughly 20% with Sanger sequencing. The mutational patterns detected by NGS are currently subjected to the same genotype interpretation systems as those detected by Sanger sequencing. However, the clinical role of drug resistance mutations between 1% and 20% prevalence is still a matter of debate and is likely different for different drugs and mutations. On the other hand, data-driven genotypic interpretation systems rely on statistical or machine-learning (ML) methods to infer drug resistance directly from data. Due to the large amount of clinical and genotypic data available, data-driven GIS have become a prominent approach to help clinicians choose effective HIV therapies, especially for heavily treatment-experienced patients with complex drug resistance patterns that have evolved over years. Early data-driven GISs, like the Virco proprietary VirtualPhenotype^TM^ (Vermeiren *et al.* 2007) and the geno2pheno system (Beerenwinkel *et al.* 2003), predict the *in-vitro* phenotype. The Virco system (VircoTYPE) was initially based on a linear regression model that estimates the phenotypic measurement as the weighted sum of the effects of individual mutations and then adapte so as to transform the predicted phenotype into clinically relevant estimate of efficacy (Winters *et al.* 2008). Geno2pheno initially assessed virus resistance to individual compounds (Geno2pheno[resistance]) and later predicted virological responses to antiretroviral (ART) regimen comprising up to four drugs. Geno2pheno-THEO estimated the probability of treatment success using genotypic data and user inputs (Altmann *et al.* 2009). Geno2pheno[resistance] (Lengauer and Sing 2006, Pironti *et al.* 2017b) uses data on viral sequence and on drugs for classification via the ML technique called support-vector machine (SVM). Geno2pheno[drug exposure] (Pironti *et al.* 2017a) uses the same input first to produce so-called drug exposure scores (DESs) correlated with prior drug exposure and secondly to supply the probabilities of exposure derived from the produced DESs to a statistical model for therapy prediction. Another software developed to infer the virological response to antiretroviral drug regimen is SHIVA which uses random forests (Riemenschneider *et al.* 2016). In one study, artificial neural networks were used for the same purpose (Larder *et al.* 2007).

Some research suggests that mutations that have been observed in the past and then disappeared from blood serum can impact a patient’s current status and the effectiveness of subsequent therapy. The time since a mutation first appeared or disappeared from blood serum may influence the degree to which that mutation informs on a patient’s status and resistance to antiretroviral drugs (Gagliardini *et al.* 2018, Ciccullo *et al.* 2021). In addition, the impact of a mutation is assumed to be more important the higher the viral load (VL) when the mutation was observed. In light of these considerations, in our model, we consider not only the mutations detected by the patient’s most recent genotypic resistance test (GRT), as in models currently in routine use. Rather, we consider the entire history of mutations recorded for the patient in the database before onset of the therapy of interest, for which we want to correctly predict the success or failure for that particular patient. Mutations are assumed to contribute additively to the patient’s resistance status. The contribution of each mutation is multiplied by a weight that incorporates (i) a degression factor for time: the further back in time the mutation was observed, the less likely it is to be still influential in determining the patient’s drug resistance, (ii) the area under the VL curve measured in a time window around the date of the mutation’s occurrence: the larger that area the more informative the mutation is considered to be, (iii) a penalty score based on the HIVdb system, in which each drug resistance mutation (DRM) is assigned a drug penalty score based on medical expertise. This score is widely recognized in the literature. Since the latent virus in organ tissue is not accessible to routine diagnostics, with this methodology, we aim to infer hidden mutations from history data on mutations detected in blood serum. Moreover, our system is based on a single support vector machines (SVM) model that predicts the therapy outcome from the patient’s mutation history.

## 2 Materials and methods

### 2.1 Problem setting

Let M={1,…,M} (*M *=* *5941) be the set of mutations considered, m∈M, denote a specific mutation, and *date_m_* the most recent point in time when that mutation was recorded in a GRT. Let D={1,…,D} be the set of drugs considered and d∈D denote an individual drug. Let $r\in {\left\{0,1\right\}}^{M}$ be a vector indicating all DRMs observed in at least one viral genotype in the set of viral genotypes considered before the onset of the therapy of interest. Eventually, the mutations this vector indicates (*r_i_* = 1) will be weighted with the weighting factors explained in the Section 2.2. Let $z\in {\left\{0,1\right\}}^{D}$ be a vector indicating the combination of drugs used in a particular therapy of interest, and let *start_z_* denote the time point of the onset of therapy *z*. Label y∈{0,1} indicates success 0 or failure (1) of the therapy. Success and failure of therapy are defined as denoted in the definition of the Standard Datum, which is given in the Section 3.2. The model is therapy-oriented, such each datapoint in the training set is composed of the *i*th *patient-therapy* pair $x_{i}=(r_{i},z_{i})$ and the corresponding value of the output *y_i_* indicating efficacy of therapy; hence it is a triple $\left(r_{i},z_{i},y_{i}\right)$. Let T={$\left(r_{1},z_{1},y_{1}),\ldots ,(r_{N},z_{N},y_{N})\right\}$ be the training set, where *N* is the cardinality of the set of patient-therapy pairs. Our goal is to train several models *f*(*x*) based on SVM that accurately predict the outcome of a target therapy *z*. The models differ in that some of them take information on the medical history of the patient into account and others do not. A comparison of the performances of models will afford insights on how relevant therapy history is for therapy prediction.

### 2.2 The weighting factors of the mutations

**Figure 1.**
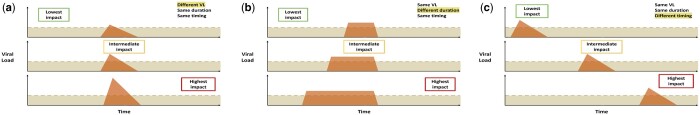
(a) How different viral loads, measured when a mutation was detected, can impact present drug resistance. (b) How different durations of a mutation can impact present drug resistance. (c) How different mutation timing can impact present drug resistance.

Previous experience suggests that the timing and duration of mutations could affect therapy efficacy. In addition, the magnitude of VL when a mutation is detected could influence viral replication. How these factors could impact the present drug resistance is shown schematically in Fig. 1. This is precisely why these factors are incorporated in the calculation of the weight factor for a mutation. In addition, the Stanford Score table is also taken into account. This is a table of scores indicating the impact of individual mutations on drug resistance of the virus. The scores for the mutations observed in a viral genotype are accumulated to yield an overall score quantifying the drug resistance of the virus to an ART regimen. The Stanford Score table is widely used as an independent predictor of virologic response to drug regimens (Tang *et al.* 2012).

**Time**. The closer the most recent time point that a mutation has been observed lies to the initiation of a drug regimen, the greater its impact on the efficacy of this regimen is assumed to be. The reason is that the latent reservoir of provirus in tissues is assumed to deplete over time. We modeled the rate of disappearance of a mutation via a sigmoid curve: for a certain period of time, the mutation has a high propensity of impacting drug resistance, then this propensity begins to decline, tending to zero. To learn the onset of the decline of the curve *α_m_* and slope *β_m_* of each sigmoid curve $y(t)=\frac{1}{1+e^{\alpha +\beta t}}$ pertaining to an individual mutation, the data on when mutations were and were not observed, respectively, were collected as explained below.

The mutations were grouped by drug class [Protease Inhibitor (PI), Nucleoside Reverse Transcriptase Inhibitor (NRTI), Non-Nucleoside Reverse Transcriptase Inhibitor (NNRTI), Integrase inhibitor (INI)]. To assess the rate of disappearance of a mutation, one has to consider periods when the patient is not on ART or is not taking any drug of a given drug class that might select for that mutation.

Consider, e.g. mutations in the PI drug class. Starting with the entire database, we consider all patients who had a therapy with at least one drug of the PI class (PI therapy) and immediately after a period when they were on another therapy that contained no drug of the PI class or a period where the patient was not under any therapy so that there was no drug pressure due to any drug from the PI class in the patient. If we consider all PI mutations present in the viral sequences during the therapy, including one or more PIs, we can see how many of those mutations disappeared at some point in one of the sequences that were sampled subsequently. If a mutation was present during the PI therapy, the following cases might occur:

- In the first sequence sampled after the PI therapy was stopped, the mutation has already disappeared. The mutation’s persistence period is assumed to be the time between the date the PI therapy was stopped and the date this sequence was sampled.
- The mutation is still present in the first sequence after discontinuing the PI therapy but disappears in the *n*th sequence observed. Here, the persistence period is the time between the date of discontinuation of PI therapy and the date of sampling of the *n*th sequence.
- The mutation is present in all subsequent sequences until the end of the period in which the patient is off any PI drug, thus we consider that the mutation has not disappeared.

Therefore, for the *m*th mutation, we construct two vectors:

- The vector *x_m_* of days elapsed from the date of discontinuation of the PI therapy to the sampling date of gene sequences at a later time point when the patient experiences no drug pressure due to drugs in the PI class (e.g. $x_{m}=[20,50,250,347,500,1000]$ would represent a sequence of six consecutive GRTs that occur on the indicated number of days after stopping a PI therapy, i.e. the first test happens on day 20, the second on day 50, etc.).
- The vector of binary values indicating whether, at each time point indicated by the vector *x_m_*, the mutation is present (1) or has not been observed (0). (e.g. $y_{m}=[1,1,1,1,0,0]$, corresponding to the vector of the previous example, indicates that the *m*th-mutation is present in the first 4 GRTs performed after therapy stop, but in the fifth test, after 500 days, is no longer there. In the data, there are also situations in which, e.g. $y_{m}=[1,1,1,0,1,0]$ where the mutation reappears.

Let $x=(x_{1},\ldots ,x_{n})$ be a random sample of *n* observations from the distribution with *probability distribution function (pdf)* $f(x;\theta )=\frac{1}{1+e^{\left(\alpha +\beta x\right)}}$ depending on the model parameter θ=(α;β). We defined the target probabilities $t_{m}=\frac{y_{m}+1}{2}$ and minimized the negative log-likelihood function (Platt 1999)

$$\min_{\theta }-\frac{1}{n}\sum (t\;\text{log}(f(x;\theta ))+(1-t)\;\text{log}(1-f(x;\theta )))$$

Equipping our statistical model for therapy efficacy with individual degression curves for each mutation is not promising, given the small amount of data we have. Thus, we decided to cluster the slope-intercept pairs of the curves for mutations pertaining to a drug class with a *k-means* algorithm after standardizing them using the *z-score*.

**Figure 2.**
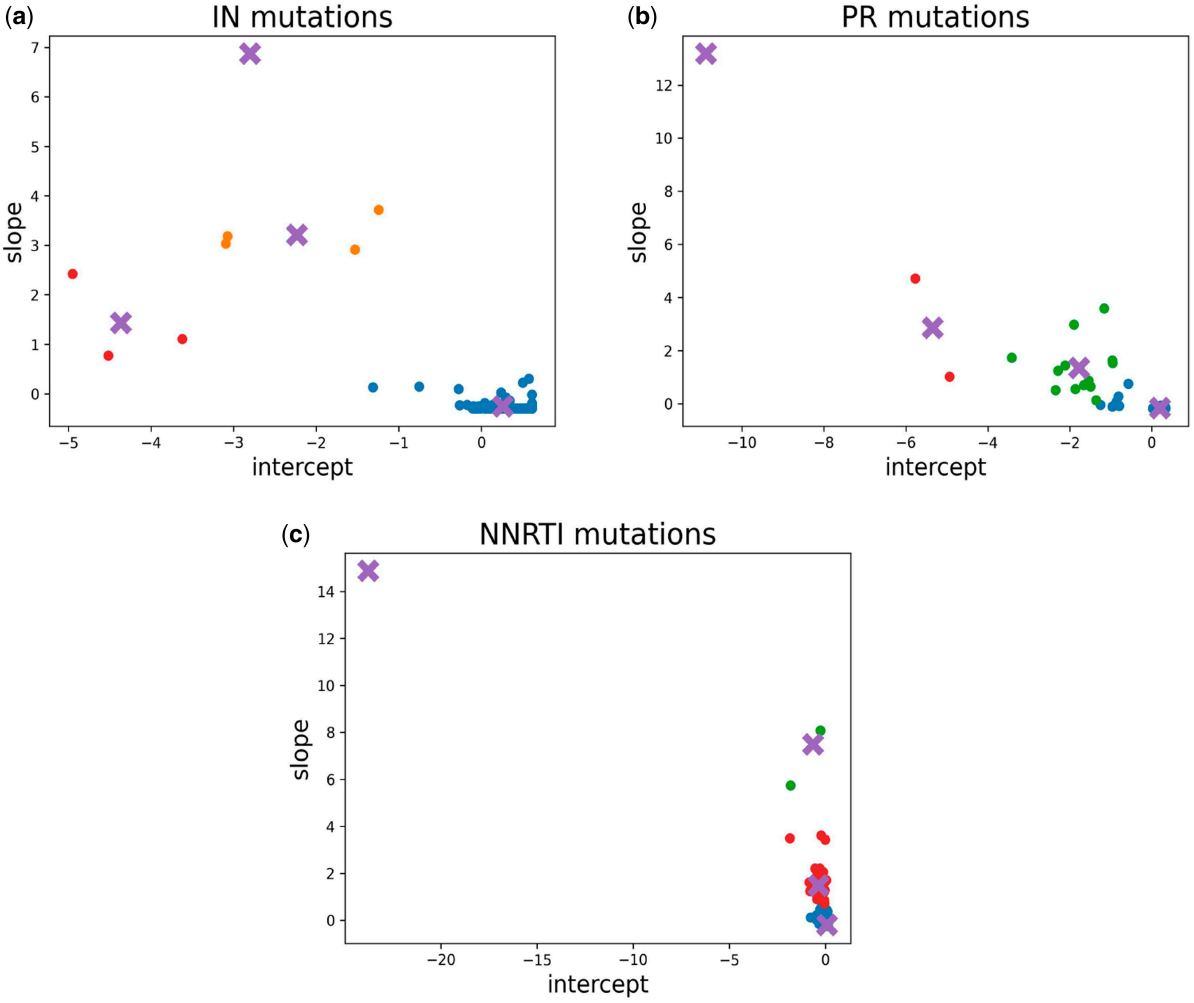
(a) Slope–intercept pairs clustered for IN-mutations, (b) slope–intercept pairs clustered for PR-mutations, and (c) slope–intercept pairs clustered for NNRTI-mutations.

This procedure was repeated for mutations pertaining to all drug classes, except for the NRTI class, because drugs from this class have been administered practically always without interruption. The clusters are depicted in Fig. 2a–c, where the points represent the slope-intercept pairs, the colors refer to the different clusters and the crosses represent the clusters’ centroids. For mutations for which no data were available to learn the slope and intercept of the sigmoid functions, the slope and the intercept of their sigmoid curves are treated as hyper-parameters of the model to be optimized by cross validation.

**Viral load**. The results in previous studies (Liu *et al.* 2022) suggest that the viral load observed in the presence of a mutation could influence the impact that the mutation has on drug resistance. To take this into account, since a value of the VL is not always present for the same day that the genetic sequence was sampled for GRT, it was decided to consider a two-month time window around the sample date and to use the area under the viral load curve obtained by performing linear interpolation of the viral load values measured within that time window. We will refer to this area as $\mathrm{Are}a_{\text{VL}}$. Note that the viral load values were logarithmized and decreased by log(50) such that VLs values below 50 *cp/ml* contribute a negative area. The rationale is that 50 is the threshold of undetectability currently used in clinical routine. For each mutation detected in the genotypic sequence of different patients, the values of $\mathrm{Are}a_{\text{VL}}$ were normalized to the interval [−1.1].

**The Stanford Score**. The Stanford score is associated with a mutation-drug pair, $s_{m,d}$. This score is the higher the more a given mutation decreases the susceptibility of the virus to the drug. Not all mutations are considered in the Stanford tables; therefore, for those for which this information is not available, the score is set to 0. If a mutation is associated with multiple drugs (across drug classes) included in the drug regimen *z_i_* and therefore multiple Stanford scores apply, the minimum Stanford score is used, because the most effective drug can be assumed to dominate the effect on the virus.

To fend against biases introduced by learning on largely differing values all scores were standardized by dividing them by the norm of the vector of all possible Stanford score s=[−15,−10,−5,0,5,10,15,20,25,30,35,40,45,50,55,60]. Hence, the Stanford score associated with a mutation is defined as follows:

$$S_{m}=\frac{\min\{s_{m,1},\ldots ,s_{m,d},\ldots ,s_{m,D}\}}{\left\|s\right\|},\;\;\text{with}\;\;d\in h$$

The weight to be given to mutation *m* is defined as follows.

(1) $$w_{m}=\frac{\mathrm{Are}a_{\text{VL}}}{1+e^{\alpha +\beta t}-\tanh(S_{m})}$$

where *t* is the time passed (in days) between the last time the mutation *m* was detected and the start of the therapy of interest *z*. That is, an increase in viral load increases the maximum weight of the mutation, and the Stanford score decreases the effect of fast and steep descent of the weight. For each mutation *m*, $w_{m}\in [-1,1]$.

To evaluate the efficacy of this approach, we trained multiple linear SVM models, contrasting the effectiveness of incorporating the patient’s complete mutational profile with baseline genotype analysis alone. In this context, we could use simple binary values or mutation-specific weights—calculated as in 1—for representing mutations in each patient-therapy sample to be given as input to the model. The choice to use linear models was dictated by the desire to maintain the interpretability of the results.

## 3 Experiments and results

### 3.1 The EuResist integrated database

The Euresist Integrated Database EIDB is one of the world’s largest databases regarding drug resistance in HIV-infected patients, both treatment-naïve and treated, undergoing clinical follow-up since 1998 (Rossetti *et al.* 2023). The data comprises nine national cohorts from Italy, Germany, Sweden, Portugal, Spain, Luxembourg, Belgium, Turkey, and Russia. Recently, data from patients referred to facilities in Ukraine and Georgia have been added. The EuResist Database was established in 2006, to collect, in an anonymized form, data on demographic and clinical characteristics of PLWH, such as antiretroviral therapies, reasons for changing therapy, treatment responses, CD4+ cell counts, AIDS-defining events, and viral co-infections.

We trained several models for predicting the success or failure of antiretroviral treatments for patients who could be either treatment-naïve or already treated. The predictors in the model include (i) the cumulative sequence of the predominant viral strains in patients’ blood collected by all genotypic resistance tests (GRTs) performed before the start of the therapy of interest, (ii) information on mutations as downloaded from the Stanford HIVDB, (iii) viral load (VL) measurements as viral RNA copies per *ml* of blood plasma (*cp/ml*), and (iv) the individual drugs used in the therapies. The response is the therapy outcome. It should be emphasized that the clinical data available on a patient is not necessarily complete.

### 3.2 The dataset

The database contains data from 105, 101 PLWH but, for many of these patients, no consistent information is available to be used for our analysis. For example, nearly 8% of patients (8, 346) do not have data on VLs, GRTs, or therapies with a valid date. Thus, in order to assemble the dataset for model training and testing, this database is preprocessed as described subsequently. Specifically, records on a subset of the patients in the dataset are selected for the analysis.

The models are *therapy-oriented*, which means that we organize the data in terms of *patient-therapy pairs*. In the context of analyzing treatment success or failure, the notion of the tuples *patient-treatment episode* (PTE) and *patient-treatment change episode* (PTCE) has been introduced (Zazzi *et al.* 2011).

**Definition patient-treatment episode**. A patient-treatment episode (PTE) consists of a genotype [amino-acid sequence of reverse transcriptase (RT), protease (PR)and/or integrase (IN)] at baseline, the set of pharmacologic compounds used in antiretroviral treatment, (cART), an optional VL at baseline, obtained no earlier than 90 days before treatment initiation, and follow-up VLs, referred to a patient. Patient-treatment episodes include both patients at first-line therapies and patient-treatment change episodes.

**Definition patient-treatment change episode**. A patient treatment change episode (PTCE) is a type of PTE. It refers to a period during which data are collected to assess how the patient responds to the change in ART that has become necessary for some reason such as virologic failure, toxicity, drug interactions, or simplification of therapy. The start of the new treatment regimen serves as the “baseline,” and the period considered is divided into two blocks, before and after start of treatment. During this episode, it is necessary to closely monitor the patient’s HIV viral load and genotype at baseline and in the follow-up period and any possible side effects.

Response to drug treatment is indicated with an outcome label y∈{0,1} indicating success or failure, respectively, according to a new EuResist Standard Datum definition that differs from the one used in the past.

**Definition standard datum**. Treatment success can be determined with a follow-up VL and optionally a VL at baseline as described. Follow-up VLs between 20 and 28 weeks after the start of therapy and the VL whose measurement date is closer to twenty-four weeks after the start of therapy are considered. Below, PTEs are referred to as successes, if the respective follow-up VL is <50 copies of HIV-1 RNA per milliliter of blood plasma. Otherwise, treatment is considered a failure. Cases in which treatment was changed before 20 weeks are considered as follows:

- Therapy lasting at most four weeks: excluded because most likely discontinued due to toxicity;
- Therapy lasting 4–8 weeks: success if a viral load below 50 cps/ml or at least 1 log decrease in the last viral load before therapy stop was observed compared with the baseline test, otherwise failure;
- Therapy lasting 8–20 weeks: success if a viral load below 50 cps./ml or at least 2 log decrease in the last viral load before therapy stop was observed compared with the baseline test, otherwise, failure.

A graphical representation of the Standard Datum is provided in Supplementary Fig. S1. The measurements of viral load have become more sensitive in recent years. Lower thresholds than previously can now be used to determine therapy success (Zazzi *et al.* 2011, 2012). Thus changing the Standard Datum accordingly in comparison with previous definitions is consistent with the current clinical practices.

In order to be included in the dataset, a patient-therapy pair must meet the following criteria:

1. The patient-therapy pair must comply with the definition of PTE, in particular, the full list of the compounds used in the therapy, at least one viral sequence observed before the start date of therapy and the follow-up VL must be present.
2. The patient that is administered that therapy must have at least one VL recorded before and after each GRT documented by a sequence at any temporal distance so that VL interpolation curves can be constructed.
3. The patient’s therapy must be able to be classified successful or unsuccessful based on the definition of the standard datum.

For each data point (patient-therapy pair), viral genotype information is provided in terms of a binary vector indicating the presence (1) or absence 0 of mutations, eventually multiplied by weights to account for the patient’s history, as described in the Section 2.2. The therapy to be administered next is also encoded by a binary vector indicating the presence or absence of the drugs appearing in the dataset. The full list of drugs considered is provided in the Supplementary material.

We consider both polymorphic and non-polymorphic mutations in our model. Polymorphisms are mutations occurring in at least 1% of viruses not exposed to selective drug pressure, i.e. reflecting natural diversity independent from therapy. A nonpolymorphic mutation does not occur in the absence of therapy (Shafer *et al.* 2007). Our decision to consider all mutations is due to the fact that there may be mutations or combinations thereof that can result in reduced susceptibility of the virus to a cART or act on the fitness of the virus, not yet recognized as such.

In the end, our dataset consists of 22 000 therapy-patient pairs, among them 12 386 successes and 9614 failures. We will refer to this dataset with the adjective *Full*. Within the *Full* dataset, we distinguish between two categories of samples: on the one hand, there are what we call type-1 therapy-patient pairs for which a detailed mutation history is available, collected through various GRTs performed prior to the start of the therapy of interest; on the other hand, there are type-2 pairs for which such a history is not available, either because they are first-line therapy or because the prior information is missing in the EIDB.

If there are no history data on a patient we also cannot leverage such data. Thus the type-2 pairs dilute our dataset in terms of the purpose of analyzing the worth of history information for therapy prediction. To address this issue we also consider what we call the *Partial* dataset which comprises type-1 pairs exclusively. The *Partial* database contains 10 581 *patient-therapy* pairs (5415 successes and 5166 failures).

From each of these two datasets—*Full* and *Partial—*two different variants were constructed, differing by the choice of which mutations to consider for each data point:

- With-History Datasets: These datasets include all mutations identified in any GRT before the therapy under consideration, allowing the complete mutation history of a patient to be exploited for analysis. These datasets are referred to with the adjective *History*.
- Without-History Datasets: These sets are limited to the mutations detected in the last GRT performed prior to therapy. These datasets are identified by the adjective *No-History*.

In summary, we have developed four variants of datasets- *Full_History*, *Full_No-history*, *Partial_History* and *Partial_No-history*-each designed for the purpose of exploring the influence of historical mutation information in treatment outcome prediction.

### 3.3 The trained models

For each dataset, two different linear-SVM models were trained, as follows:

- *Non-weighted* models: mutations are treated in a binary fashion. That is present (1) or absent (0).
- *Weighted* models: mutations are not represented in binary but weighted with the degression weight introduced above, offering varying degrees of significance or prevalence of mutations.

**Figure 3.**
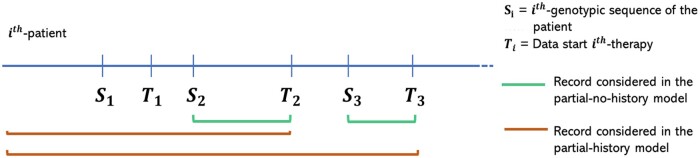
Therapies and relative patient history considered in the history model and in the no-history model.

The models trained will be referred to as *(Full/Partial)_(History/No-history)_(Weighted/Non-weighted)* models. The qualifiers *History*, *No-history*, *Weighted* and *Non-weighted* refer to the type of dataset used for training and testing and to the choice to weight or not to weight the mutations. Supplementary Table S2 presents a schematic view of the types of models we trained. Figure 3 illustrates an example of the therapies considered in the *Partial_History* and *Partial_No-history* models, respectively. Therapy *T*_1_ is not considered because prior to that therapy we have only one genotype at baseline.

### 3.4 Experimental setting

The *Full* datasets are slightly unbalanced: 56.3% of the records pertain to successes. For the *Partial* datasets, the respective fraction is 51.17%.

The *Full* datasets are slightly unbalanced: 56.3% of the records pertain to successes. For the *Partial* datasets, the respective fraction is 51.17%.

The data were randomly divided into training set (75%) and test set (25%). To avoid data leakage, therapies belonging to the same patient were included in the same set. A linear Support Vector Machine for classification was trained to predict success, or failure labels based on outputs representing probability estimates (Platt 1999). A random search of the hyperparameters was carried out in order to determine the parameters for the best possible prediction performance. Values of hyperparameters were sampled from a probability distribution, and the performance of the resulting models was evaluated by a five times 5-fold cross-validation. In this way, for each parameter setting, we obtain 25 performance values of the model with that setting when using different training and validation sets. In particular, the regularization parameter *C* was sampled from a log-uniform distribution $\left(C∼U(e^{-14},1)\right)$. For each cross-validation set, the model with the lowest value of *C* whose average performance (in terms of ROC-AUC score) was not significantly lower than the best average performance was selected [Benjamini–Hochberg-corrected Wilcoxon signed-rank test (Wilcoxon 1945) with a significance threshold of 0.05]. For the mutations for which no data was available to fit the parameter *θ* of the sigmoid curve, *θ* was treated as a hyperparameter of the model, sampled uniformly between the minimum and the maximum value of the parameters *α* and *β* learned for the mutations of the same drug class.

### 3.5 Results

Model performances were compared performing the Nadeau and Bengio statistical test (Nadeau and Bengio 1999) on ROC-AUC scores, with a significance level of 5%.

***Partial_model* results.** The results obtained from the two models based on the partial datasets are shown in Table 1. The *Partial_History_Weighted model* achieves almost two percentage points more in ROC-AUC score than the *Partial_No-history_Non-weighted model*, exhibiting a statistically significant difference between the two approaches.

**Table 1.** Performance metrics of models trained with the Partial dataset.^a^

Model	AUC	Acc	Rec	Spec
*Partial_History_Weighted*	**72.42**	**67.45**	61.85	**72.17**
	(±0.140)	(±0.133)	(±0.196)	(±0.166)
*Partial_No-history_Non-weighted*	70.43	65.33	**62.84**	67.43
	(±0.139)	(±0.129)	(±0.204)	(±0.165)
	0.0011*			

* The *P*-value w.r.t. the history_weighted model.

a The metrics reported are ROC AUC score (AUC), Accuracy (Acc), Recall (Rec) and Specificity (Spec) in percentage (%).

Best results highlighted in bold.

***Full_model* results.** Table 2 shows the results obtained using the four models based on the full datasets. Thanks to the availability of more data points, the models obtain better results overall compared to the Partial models. In particular, the *Full_History_Weighted model* reaches the highest ROC-AUC score of 76.34% (±0.099). The *P*-values associated with the ROC-AUC scores of the models that do not account for or partially account for historical information when compared with the *Full_History_Weighted model* show that the difference in performance is statistically significant.

**Table 2.** Performance metrics of models trained with the Full dataset.^a^

Model	AUC	Acc	Rec	Spec
*Full_History_Weighted*	**76.34**	**70.74**	64.95	73.28
	(±0.099)	(±0.087)	(±0.148)	(±0.010)
*Full_No-history_Weighted*	76.13	70.60	**65.31**	72.80
	(±0.099)	(±0.088)	(±0.151)	(±0.101)
	*0.0064			
*Full_History_Non-weighted*	76.67	70.10	58.32	**76.58**
	(±0.100)	(±0.090)	(±0.147)	(±0.101)
	1.02e−32*			
*Full_No-history_Non-weighted*	74.98	69.60	58.41	76.01
	(±0.098)	(±0.088)	(±0.144)	(±0.102)
	6.92e−23*			

* The *P*-value w.r.t. the history_weighted model.

a The metrics reported are ROC AUC score (AUC), Accuracy (Acc), Recall (Rec) and Specificity (Spec) in percentage (%).

Best results highlighted in bold.

Additional statistical analysis was conducted to assess the impact of incorporating historical information. Specifically, an attempt was made to compare the performance of the *Full_history_weighted* (H) model and the *Full_No-history_Non-weighted* (NH) model. This comparison aimed to evaluate the influence of historical information on the predictive accuracy of the models. Table 3 presents the mean and standard deviation of predicted probabilities for treatment successes and failures, ranked according to whether or not historical information was considered. Notably, when only treatments with a history (indicated with a ✓) are considered, i.e. type-1 therapy-patient pairs, the H-model consistently outperforms the NH-model in predicting the probabilities of both success and failure. This suggests that incorporating historical information provides valuable insights for accurate prediction.

**Table 3.** Mean and standard deviation of predicted probabilities by the two models, divided by type of therapy (success or failure).^a^

Type_of therapies correctly classified	Type_of model	Only therapy with history	Mean±SD_of predicted probabilities
Successes	H	✓	0.720 ± 0.11
Successes	NH	✓	0.699 ± 0.20
Failures	H	✓	0.699 ± 0.12
Failures	NH	✓	0.677 ± 0.099
Successes	H	×	0.724 ± 0.106
Successes	NH	×	0.700 ± 0.1146
Failures	H	×	0.667 ± 0.119
Failures	NH	×	0.665 ± 0.098

a The column “only therapy with history” is valued with ✓ when only therapies with more than one previous genotype are considered or with × when all the therapies are considered.

Wilcoxon tests were performed on the probability distributions of treatment outcomes between the H-model and NH-model to assess the statistical differences between the probabilities predicted by the two models. The respective null hypotheses are given in Table 4. Results show significant differences between the H and NH models. For both failures, with and without historical information, the H-model outperforms the NH-model, as evidenced by the remarkably low *P*-values ($5.32e^{-16}$ and $9.03e^{-25}$, respectively). Regarding all treatment successes, the H-model significantly outperforms the NH-model, with a *P*-value of $7.15e^{-6}$. However, when narrowing down the analysis to only successes with historical information, the high *P*-value (0.9536) indicates that the null hypothesis that the H-model predicts smaller or equal probabilities than the NH-model cannot be rejected. This can be interpreted to indicate that historical information does not play a significant role in the prediction of successful therapies.

**Table 4.** P-values of the Wilcoxon tests performed on the predicted probability distributions of the models, with the null hypothesis *H*_0_.

Wilcoxon test Between H and NH probability distribution for:	*P*-value
Successes	**7.15e^–6^**
$H_{0}:p(\mathrm{Succes}s_{H})\leq p(\mathrm{Succes}s_{NH})$	
Successes with history	0.9536
$H_{0}:p(\mathrm{Succes}s_{H})\leq p(\mathrm{Succes}s_{NH})$	
Failures	**5.32e^–16^**
$H_{0}:p(\mathrm{Failur}e_{H})\leq p(\mathrm{Failur}e_{NH})$	
Failures with history	**9.03e^–25^**
$H_{0}:p(\mathrm{Failur}e_{H})\leq p(\mathrm{Failur}e_{NH})$	

In bold p-value < 0.05 that indicates rejection of the null hypothesis.

**Figure 4.**
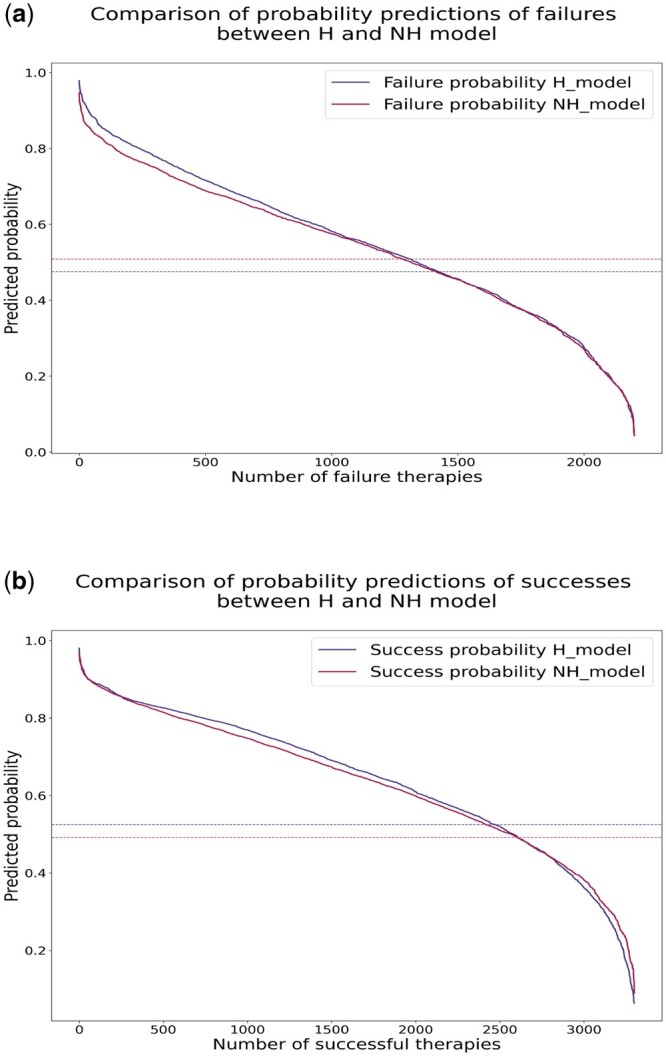
Plots of the probability distributions predicted by the models, respectively (a) for successes and (b) for failures. On the *x*-axis, there is the number of therapies, and on the *y*-axis, the models’ predicted probabilities. The blue and red dotted lines represent the cut-offs for probabilities, respectively, for the H and NH-model. For each model, the portion of the line above its cut-off represents therapy correctly classified by the model, while the one below represents therapy incorrectly classified by the model.

Figure 4 displays the H and NH-model’s distributions of predicted probabilities for successes and failures. For both models, the cut-offs for probabilities are represented for both models by lines parallel to the *x*-axis. For ease of identification, each cut-off line is colored to match the color of its associated model. These cut-offs for probabilities are the threshold values that determine the class assignment for each data point based on the probabilities predicted by the models, and they have been tuned as reported in the Supplementary material. For each model in the figure, the part of the line above its cut-off represents successful or failing therapies correctly classified by the model, while the portion below shows therapies incorrectly classified. In general, the H-model seems to predict higher probabilities than the NH-model. Focusing on failures, Fig. 4a shows that the H-model correctly classifies more failures than the NH-model. Regarding the space between the two cut-offs, where failures are correctly classified by the H-model but not by the NH-model, the H’s probabilities are slightly higher. Curve segments below the lower cut-off represent failures misclassified by both models, with comparable probability distributions. Focusing on successes, Fig. 4b shows fewer successes classified correctly by the H-model than the NH-model. However, the probabilities predicted by the H-model are higher, even for successes only correctly classified by the NH-model. NH-model’s probabilities are higher for misclassified successes represented by lines below the lower cut-off. These data support the intuition that mutation profiling for each patient is useful for considering past resistance that may still play a role when changing therapy is necessary, to avoid failure.

## 4 Discussion

We have conducted several statistical analyses in order to assess the impact of incorporating historical information into the prediction of the efficacy of anti-HIV drug therapy.

Models using *Partial* datasets, including only patient-therapy pairs with relevant medical history before the target therapy (type-1 pairs), exhibit significant differences in prediction performance. These underscore the importance of incorporating a patient’s history into predictions of treatment outcomes, especially when eliminating the data dilution associated with therapies that lack the previous history.

Deeper analyses were conducted on the *Full* models for the importance of training on *Full* datasets. This includes both patient-therapy pairs with relevant medical history before the target therapy and patients without historical information. The latter include patients for which limited data are available or those on first-line therapy. Ensuring accurate predictions for both categories is essential for providing valuable insights for clinical decision-making. The *Full_History_Weighted model* (H) model has a higher ROC-AUC score (76.34%) than the *Full_No-history_No-weighted model* (NH) (74.98%). Interestingly, the *Full_No-history_Weighted model* demonstrates performance comparable to the *Full_History_Weighted model*. Both the models take historical information into account to some extent: the former because it considers the mutations of all the GRTs performed in the past and weighted as explained earlier; the latter because, although only the last GRT is considered, the mutations are weighted taking into account the elapsed time since the mutation was detected, that is the date on which the virus was sequenced. This suggests that the substantial improvements observed with the history-weighted model can be attributed mainly to incorporating the weighting factors rather than including historical mutations *per se*.

One reason why the H-model has better predictive performance than the NH-model, especially in the case of failures, could be attributed to the possibility that historical mutations represent latent reservoirs for HIV. Latent reservoirs are cells infected with the virus but in tissues other than blood serum. These remain inactive or dormant, evading the body’s immune response to standard ART. The existence of latent reservoirs makes it difficult to eradicate HIV from the body because these cells can reactivate and generate new viral particles, leading to a reappearance of the virus and treatment failure. Presumably, the model, considering weighted historical mutations, can capture the intricate relationship between latent reservoirs and response to therapy, improving predictive performance in case of treatment failure.

Our study’s implications align with a concept emerging from a recent study conducted by part of our team to study mutational history in a different context (Pirkl *et al.* 2023). In this work, data from various patients are used cross-sectionally rather than longitudinally to infer the accumulation of mutations in multidrug-resistant patients, i.e. the order in which mutations occur over time (mutational history). Therefore, the respective model can predict aspects of mutational history from current genotypes. For example, if we observe a genotype with the X mutation and not with the Y mutation, but in the mutational history, Y was observed before X, Pirkl *et al.* (2023) hypothesize that the Y mutation may now be present only in the latent reservoir genotypes and not in the blood serum and that mutations are still present in the latent reservoir and thus have an impact on future therapies. Both studies emphasize the importance of mutations that occurred in the past for future therapies and drug resistance, respectively.

Additional experiments with the prediction of treatment outcome have been carried out using the Stanford Treatment Change episode database available at https://hivdb.stanford.edu/TCEs/. The results are reported in the section *Additional results* of the Supplementary material and confirm the insights on the importance of historical mutations.

In conclusion, our study sheds light on the fact that incorporating the temporal dynamics of virus acquiring mutations in response to drug therapy improves prediction accuracy compared with the standard analysis of only the last available genotype. The results underscore the importance of considering mutation dynamics and its potential influence on treatment outcomes. They provide valuable insights into the complex dynamics of HIV infection, guiding future research and informing the development of effective therapeutic strategies.

Limitations of our work include the potential suboptimality of the constructed weighting factor for mutations, which combines VL magnitude, mutation timing, and duration, without a clear understanding of the individual influences of these three aspects. Furthermore, although the differences in performance between the models that include history and those that do not are statistically significant, the ROC-AUC of the non-history models is relatively high. This could be due to the methodology used to include history or the inherent incompleteness of the available data.

Our findings can be interpreted in two ways. On the one hand, the fact that historical mutations impact therapy prediction in a statistically highly significant fashion points to the importance of involving mutational history in the research on therapy prediction. Of note, this implies continuing to consider past data when investigating response to novel and future treatments as a general research plan. On the other hand, the fact that the impact is quite small in absolute terms means that the clinical relevance of incorporating historical mutations in the analysis of viral resistance for an individual patient is debatable. This can be viewed as support for alleviating the clinician from the need to elaborate on and input complex data into a genotypic prediction system, although reasoning on past treatment and resistance data remains well established in the clinician’s choice of a new therapy.

## Supplementary Material

btae327_Supplementary_Data

## Data Availability

This analysis was conducted using the EIDB. For further validation, we encourage reproducing this study with the latest release of the EIDB, which can be accessed upon request through the Euresist Network at https://www.euresist.org/become-a-partner. Due to the sensitive nature of personal medical data, it is not feasible to make this data publicly available on the internet. Additionally, data from the HIVdb were utilized in this study. The HIVdb is openly accessible at https://hivdb.stanford.edu/TCEs/.
