# Supplementary material for: Incorporating temporal dynamics of mutations to enhance the prediction capability of antiretroviral therapy’s outcome for HIV-1
Source: Bioinformatics. 2024 May 22;40(6):btae327. doi: 10.1093/bioinformatics/btae327 (PMC11153833; doi:10.1093/bioinformatics/btae327)
Supplement: btae327_Supplementary_Data [file btae327_supplementary_data.pdf]

Supplementary material

A. Details on the datasets and on results

The drugs considered in the datasets are the following: lamivudine (3TC), abacavir (ABC), amprenavir (APV), atazanavir (ATV), zidovudine (AZT), bictegravir (BIC), cabotegravir (CAB), stavudine (D4T), zalcitabine (DDC), didanosine (DDI), delavirdine (DLV), doravirine (DOR), darunavir (DRV), dolutegravir (DTG), efavirenz (EFV), etravirine (ETR), elvitegravir (EVG), fosamprenavir (FPV), emtricitabine (FTC), indinavir (IDV), lopinavir (LPV), nelfinavir (NFV), nevirapine (NVP), raltegravir (RAL), rilpivirine (RPV), saquinavir (SQV), tenofovir alafenamide (TAF), tenofovir disoproxil (TDF), tipranavir (TPV).

Due to the definitions of PTEs (Patient-Treatment Episodes) and PTCEs (Patient-Treatment Change Episodes), it is feasible for various entries in the datasets, involving distinct treatments, to relate to a single patient undergoing different therapies in different time periods. Table 1 reports how many therapies there are for the same patient, divided between successful (label 0) and failed therapies (label 1), according to the Standard Datum definition provided in the main text, in Section 3.2. A graphical representation of the Standard Datum is given in Figure 1. To guard against data leakage, either all therapies referring to the same patient were included in the training set or all of them were included in the test set.

Four different datasets have been built and six different linear-SVM models have been trained. A tabular form of listing the various models and datasets used in a simple visualization is presented in Table 2.

# Therapies per patient	Label	# Patients
1	0	4659
1	1	3363
2	0	1781
2	1	1230
3	0	691
3	1	501
4	0	253
4	1	235
5	0	96
5	1	118
6	0	44
6	1	49
7	0	19
7	1	26
8	0	14
8	1	14
9	0	4
9	1	10
10	0	5
11	1	3
11	1	1
12	1	2
15	1	1

Table 1. Number of therapies per patient

A.1. Significance test

To assess whether the history model performs better than the no-history model in terms of generalization capability, we use significance tests. The most common method for comparing the performance of Machine Learning models is the paired Student's t-test combined with random subsampling of the training set. However, one of the key assumptions of this test is that the underlying data be sampled independently from the two populations being compared. Since the models are trained on the same training set, the paired Student's t-test could lead to misleading results, with a high false positive rate (i.e., having a high probability of rejecting the null hypothesis indicating that the two models are significantly different, when this is in fact the case, i.e. overrating the significance of using history data). Nadeau and Bengio (Nadeau and Bengio, 1999) propose a variance correction that accounts for the dependence between the two. We apply their significance test in our analysis, with a significance level of 5% .

A.2. Cut-off for the predicted probabilities

The trained models generate probabilities that indicate the likelihood of belonging to a certain class. Typically, a default threshold of 0.5 is used to assign class labels based on these probabilities. However, relying solely on this default threshold can lead to suboptimal performance, particularly when dealing with datasets that have unbalanced class distributions. Although, in our case, the overall dataset is not highly unbalanced, it is still helpful to adjust the threshold according to the characteristics of the training data.
A simple strategy was used to determine the most suitable threshold for mapping probabilities onto class labels. A comprehensive search was conducted with 1,000 threshold values equally spaced between 0 and 1. Through this iterative process, each model identified an individual threshold that maximized balanced accuracy. This ensures that the threshold is tailored to the specific characteristics of each model, finding a desirable compromise between sensitivity and specificity.
Through this iterative threshold selection approach, we improve model performance and obtain more accurate classification results. It allows us to effectively handle variations within the dataset and address situations where imbalances between classes may affect the model's predictive capabilities.

B. Analysis of the importance of mutations

B.1. Mutations ranking

Identifying the role of mutations in predicting the outcome of therapy is paramount for various reasons. The response of HIV to specific antiretroviral drugs can vary depending on the mutations present in its genome. Gaining an understanding of the role of mutations is crucial for improving treatment recommendations and maximizing treatment effectiveness. It enables us to identify mutations associated with drug resistance and to comprehend how these mutations interact and impact viral suppression. This knowledge aids in selecting appropriate drug regimens that optimize treatment outcomes.

The model trained is a linear Support Vector Machine. The linear SVM provides hyperplane coefficients that represent the weights assigned to the features of the input data. These coefficients serve for assessing the importance and contributions of each feature in the SVM's decision-making process. The higher the absolute value of a mutation's coefficient, the

Model name	Possible # of GRTs > 1 prior to the target therapy	# of GRTs = 1 prior to the target therapy	Mutations of the GRT at baseline	The mutations of GRTs before the one at baseline	Binary vector of mutations	Weighted vector of mutations
Partial_History_Weighted	✓	×	✓	✓	✓	✓
Partial_No-history_Non-weighted	✓	×	✓	×	✓	×
Full_History_Weighted	✓	✓	✓	✓	✓	✓
Full_No-history_Weighted	✓	✓	✓	×	✓	✓
Full_History_Non-weighted	✓	✓	✓	✓	✓	×
Full_No-history_Non-weighted	✓	✓	✓	×	✓	×

Table 2. Datasets and models' characteristics

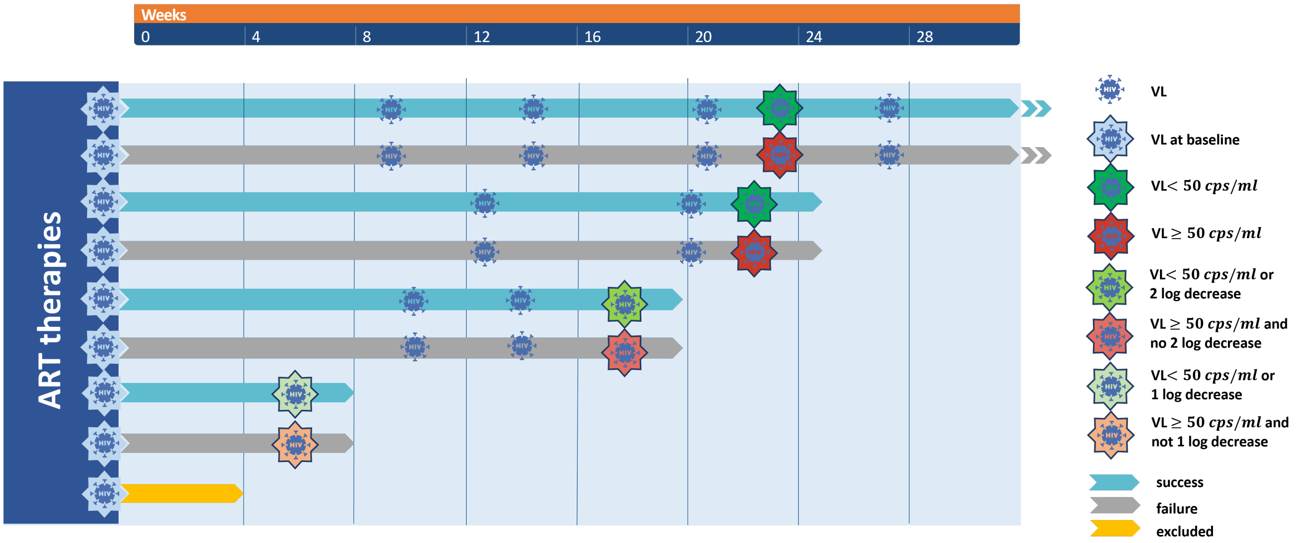

Fig. 1. Graphical representation of how to label an ART therapy as success or failure according to the Standard Datum definition.

more that mutation plays a role in determining the outcome. Moreover, we calculated individual weights for each mutation of each patient, as described in the main paper in Section 2.2. If a mutation is assigned a low weight, at least one of the following conditions occurs: *(i)* it was observed far back in time, *(ii)* the patient's viral load, at the time of observing the mutation, was a low level or undetectable, *(iii)* the Stanford score associated with the mutation is low. The low weight associated with a mutation suggests that the mutation does not highly impact therapy outcome.

In studying the impact of mutations, our objectives were twofold:

- To identify the mutations that consistently have a significant impact on therapy outcome.
- To identify specific mutations that are assigned a high model coefficient despite entering the model with a low weight.

For the first objective, we simply ranked of mutations according to the absolute values of their SVM coefficients. Inspecting Table 3 we can make interesting observations. For example, the top-ranking mutation is T200K. This mutation is not widely recognized as an important mutation for drug resistance in HIV but a few studies reported that T200K, even though it had not been reported previously, might be associated with NVP resistance (Hachiya et al., 2004). In general, many of the top-ranked mutations in Table 3 are not recognized resistance mutations. Their coefficient values may be high because they were frequently observed near the start of the therapy of interest (i.e., the most recent genotype) and/or in the presence of a high viral load. Whereas most of the literature focuses on mutations that reduce viral fitness as something potentially exploitable in the clinic, these mutations could instead increase viral fitness and, thus, viral load.

For the second objective, a ranking formula was created to consider the potential discrepancy between the SVM coefficient and the customized weights, allowing mutations with high coefficients but low weights to still receive a significant ranking value. The ranking formula for each mutation m is the following:

$$\text{Ranking\_value}_m = |\text{coefficient}_m|_{\text{z\_scaled}} \cdot \Big(-\sum_{i=1}^{n} \log w_m^i\Big)_{\text{z\_scaled}} \qquad (1)$$

where *coefficient* is the SVM hyperplane coefficient, n is the size of the training set and w_m^i is the weight learned

for mutation m of therapy-patient tuple i. We obtained the positional ranking of the mutations by decreasingly sorting the absolute $Ranking\_value$ for each mutation. From this ranking, we identify 149 mutations that could have the impact we aim for, which are the ones on the left of the dotted red line, representing the flexion point of the curve, in Figure 2a. That is, we applied the elbow method on the scree plot in the figure. The top-ranking 30 mutations are listed in Table 4. Some observations considering this ranking may be clinically relevant. For example, L63P, among the top-ranked mutations, is usually not considered a resistance mutation. However, it has been pointed out that *(i)* this mutation may persist for even more than 18 months despite therapy changes (Pao et al., 2004), *(ii)* the mutation by itself does not render the virus resistant to inhibitors, but it does help the virus replicate more effectively, especially when under pressure from drugs. This suggests that small changes in the virus can have significant compensating effects, which could contribute to the evolution of drug-resistant variants of HIV-1 (Suñé et al., 2004). Indeed, most of mutations included in Table 4 that are not considered resistance mutations have been implicated in compensatory or resistance modulation mechanisms. In PR, L10I, L63P, I93L even had a Stanford HIVdb score (although low) in past versions. The same applies to RTE44D and RTH208Y which have been studied in detail (Betancor et al., 2014; Girouard et al., 2003; Nebbia et al., 2007; Romano et al., 2002). Being aware that mutations that are not considered major could increase viral fitness and have a compensatory effect that helps the virus replicate more effectively while not making the virus resistant to inhibitors per se, could have important implications for clinical practice. In particular, rule-based genotypic interpretation systems that consider only major resistance mutations may not be sufficient to design a therapy that considers all factors that play a role in the long-term efficacy of therapy.

B.2. Enrichment analysis

An enrichment analysis of the mutations was performed. Enriched mutations are those that have become more widespread or common within a particular population or sample compared to their initial occurrence. The implication of enriched mutations could be *(i)* drug resistance; *(ii)* disease progression: independent of drug resistance, such as through tropism change or increased virulence.

To perform this analysis, Stanford tables on https://hivdb.stanford.edu/cgi-bin/MutPrevBySubtypeRx.cgi were used to analyze whether the frequency of each mutation of interest increased from naïve patients to treatment-experienced patients. Specifically, at the linked website, each table presents the results of an enrichment analysis on the genotype sequences of patients treated with PI, or NRTI, or NNRTI as first-line therapy. Columns specify HIV subtypes (A, B, C, D, F, G, AE, AG), while rows detail mutations by amino acid deviating from consensus B. We focused our analysis on the HIV B subtype column, as it has the largest number of sequences available, allowing for a more robust and representative mutation enrichment analysis. The intersection between a mutation row and a subtype column highlights the frequency of patients with that mutation for the specified subtype. Where the frequency is greater than 1% indicates an enriched mutation that therefore could be clinically or biologically significant. Of the 149 mutations identified in Section B.1, we counted how many are present in the tables just described. It resulted that 62% of the 149 selected mutations become enriched. This confirms that the approach we used in this work in treatment-experienced patients captured mostly biologically meaningful mutations, either conferring or modulating resistance. Importantly, one-third of the scored mutations do not appear to be enriched following treatment but may play a role for the clinical outcome. Mutations or polymorphisms of this kind may be associated with functional constraints limiting their selection under commonly used therapies yet play a role in response to treatment because of effects that are independent of drug resistance, e.g., impact on innate or adaptive immunity and fitness effects.

Mutation	Value coefficient H model
RTT200K	0.3207
RTD113N	0.2964
INK14R	0.2819
RTT200V	0.2536
RTS322A	-0.2513
INM154I	-0.2466
RTK249R	-0.2434
RTK122Q	-0.2390
PRL63C	0.2278
RTM16V	-0.2258
RTE79K	-0.2235
**PRV82S**	0.2165
RTV245I	-0.2145
RTQ145E	0.2136
RTQ207H	-0.2095
PRG17D	0.2090
PRH69Y	0.2068
RTP176S	-0.2058
PRI93L	-0.1999
**RTF227L**	0.1977
RTS48P	0.1960
**RTT215Y**	0.1959
RTY271F	0.1950
RTE194D	-0.1939
RTV35E	0.1919
**PRL90M**	0.1914
RTL210S	0.1885
RTE248N	-0.1882
PRL19T	0.1878
INV37I	0.1862

Table 3. First 30 mutations of the ranking obtained ordering the mutations with descending absolute value of H model coefficients. The mutations name is preceded by PR (protease), RT (reverse transcriptase) or IN (integrase), depending on the region of the HIV genome. In bold, Stanford mutations.

C. Additional results

In this section, we present additional computational results obtained when using the Treatment Change Episode (TCE) repository available from the Stanford University HIV drug resistance database (https://hivdb.stanford.edu/TCEs/). The TCE Repository presents over 1,500 cases of TCE. Each TCE is coded through an XML schema that includes key clinical information including *(i)* previously administered ARTs; *(ii)* plasma HIV-1 RNA load, CD4 cell counts, and results of genotypic resistance assessments at baseline; *(iii)* administration of subsequent salvage therapies; and *(iv)*

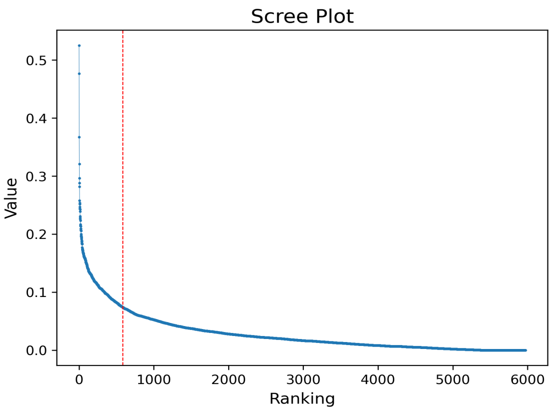

(a)

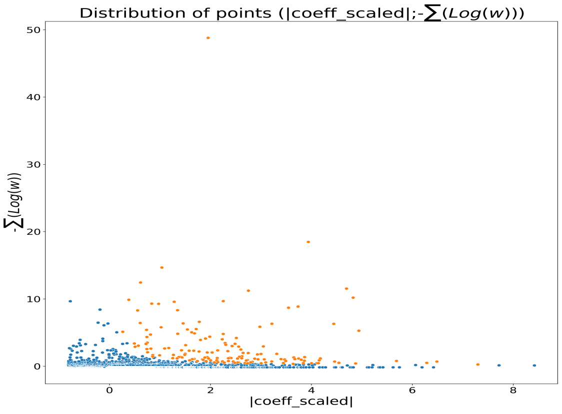

(b)

Fig. 2. Figure (a) is the scree plot of the mutation, based on the ranking values obtained by equation 1. Figure (b) is the scatter plot of the points ($|coefficient_m|_{z\_scaled}; -\sum_{i=1}^{n} \log w_m^i$) to show that the ranking does not depend only to one of the two factors of the ranking formula but by both of them. The orange points are the mutations identified in the scree plot before the dashed red line that is the flexion point of the curve.

Mutation	Ranking value
**RTM184V**	95.30
**RTK103N**	72.80
**PRL90M**	54.19
**RTT215Y**	49.21
**RTK65R**	33.17
RTD123E	30.88
**RTK70R**	30.86
PRL63P	28.02
PRI93L	26.13
RTK122E	21.81
**RTM184I**	20.33
**RTK219Q**	17.46
**PRI84V**	15.21
**RTD67N**	12.26
RTK277R	11.74
RTG196E	11.23
RTE44D	10.82
RTK49R	10.58
**RTT215F**	9.50
**RTL210W**	9.28
PRL10I	9.00
RTP294T	8.99
**PRV82A**	8.94
**RTL100I**	8.74
RTI202V	8.43
RTS162C	8.32
RTI293V	8.24
RTT286A	8.20
RTH208Y	7.88
PRI64V	7.81

Table 4. First 30 mutations of the ranking obtained ordering the mutations with descending ranking value computed as in 1. The mutations name is preceded by PR (protease), RT (reverse transcriptase) or IN (integrase), depending on the region of the HIV genome. In bold, Stanford mutations.

measurements of plasma HIV-1 RNA levels made during the course of salvage therapy.

A key pre-processing step was implemented to harmonize the Stanford TCE data with those from the EIDB, for both structure and information content, ensuring consistency and compatibility within the experimental analysis. This procedure involved a selection of PTEs that *(i)* met the specific criteria for evaluating therapeutic outcomes, as outlined in the Standard Datum definition within Section 2 of the main paper, and *(ii)* had enough information to determine mutation weights, as explained in Section 3.2 in the main paper. This resulted in the identification of a restricted dataset of 562 PTEs.

In this dataset, only 63 PTEs (11.2%) are labeled as successes. There are two main reasons why this dataset is heavily skewed toward treatment failures. First, data collection may not have placed emphasis on creating a representative sample of HIV treatments. When asked to contribute to the TCE repository clinicians may simply have provided available genetic sequences, leading to an over-representation of genotypic tests done in conjunction with treatment failures because this has been the most typical use of genotyping for many years, particularly when treatment failure was common. Second, due to timing of the request to contribute to the TCE repository, the data refers to ART administered before 2011, i.e. before widespread use of the most effective treatment regimens, including second-generation INSTI therapies. This resulted in the collection of ARTs with significantly higher failure rates than more recent treatments, further exacerbating the dataset's skew toward therapy failures.

The Stanford TCE dataset was employed in two distinct ways:

- As an external validation set for testing models already trained on the EIDB-derived datasets. The results are shown in Table 5. Although this analysis does not yield results as conclusive as those obtained on the EIDB-derived test sets presented in Table 2 of the main paper, they point the same direction. The loss in the model performance is mainly due to the unbalanced distribution of the TCE dataset, which is skewed toward cases of treatment failure, unlike the dataset used for model training. Nevertheless, the analysis confirms that incorporating the temporal dynamics of mutations improves prediction accuracy compared with the standard analysis of the last available genotype, helping more in the prediction of failures than successes.

Model	AUC	Acc	Rec	Spec
Full_History_Weighted	66.1 (±0.54)	89.8 (±0.16)	94.2 (±0.16)	55.5 (±0.65)
Full_No-history_Weighted	58.7 (±0.52) *7.32e-62	79.0 (±0.23)	85.2 (±0.25)	50.8 (±0.60)
Full_History_Non-weighted	64.0 (±0.54) *3.94e-7	77.2 (±0.22)	80.0 (±0.24)	55.5 (±0.74)
Full_No-history_Non-weighted	57.7 (±0.53) *2.45e-73	73.7 (±0.23)	77.0 (±0.25)	47.6 (±0.68)

*indicates the p-value w.r.t. the history_weighted model

Table 5. Performance metrics of models on the external validation set derived from Stanford TCE dataset, when models are trained on the EIDB. The metrics reported are ROC AUC score (AUC), Accuracy (Acc), Recall (Rec) and Specificity (Spec) in percentage (%).

Model	AUC	Acc	Rec	Spec
Full_History_Weighted	76.2 (±1.10)	77.1 (±0.49)	78.1 (±0.52)	66.7 (±2.00)
Full_No-history_Weighted	66.1 (±1.33) *6.27e-04	65.7 (±0.57)	66.4 (±0.55)	58.3 (±2.3)
Full_History_Non-weighted	73.4 (±1.01) *3.78e-02	72.9 (±0.51)	75.0 (±0.52)	50.0 (±2.23)
Full_No-history_Non-weighted	71.1 (±0.99) *2.75e-03	72.1 (±0.55)	71.9 (±0.50)	75.0 (±1.9)

*indicates the p-value w.r.t. the History_weighted model

Table 6. Test set performance metrics of models trained on the Stanford TCE dataset. The metrics reported are ROC AUC score (AUC), Accuracy (Acc), Recall (Rec) and Specificity (Spec) in percentage (%).

- As a new dataset to be divided into training set and test set to train and test the *Full* models. The results are shown in Table 6. The statistically significant differences between the AUC of the models compared to the *Full_History_Weighted_model* confirm the importance of considering the mutation history of each patient when evaluating the administration of a new ART.

References

Betancor, G., Nevot, M., Mendieta, J., Gómez-Puertas, P., Martínez, M. A., and Menéndez-Arias, L. (2014). Molecular basis of the association of H208Y and thymidine analogue resistance mutations M41L, L210W and T215Y in the HIV-1 reverse transcriptase of treated patients. *Antiviral Res.*, 106:42–52.

Girouard, M., Diallo, K., Marchand, B., McCormick, S., and Götte, M. (2003). Mutations E44D and V118I in the reverse transcriptase of HIV-1 play distinct mechanistic roles in dual resistance to AZT and 3TC. *J. Biol. Chem.*, 278(36):34403–34410.

Hachiya, A., Gatanaga, H., Kodama, E., Ikeuchi, M., Matsuoka, M., Harada, S., Mitsuya, H., Kimura, S., and Oka, S. (2004). Novel patterns of nevirapine resistance-associated mutations of human immunodeficiency virus type 1 in treatment-naïve patients. *Virology*, 327(2):215–224.

Nadeau, C. and Bengio, Y. (1999). Inference for the generalization error. In Solla, S., Leen, T., and Müller, K., editors, *Advances in Neural Information Processing Systems*, volume 12. MIT Press.

Nebbia, G., Sabin, C. A., Dunn, D. T., Geretti, A. M., UK Collaborative Group on HIV Drug Resistance, and UK Collaborative HIV Cohort (CHIC) Study Group (2007). Emergence of the H208Y mutation in the reverse transcriptase (RT) of HIV-1 in association with nucleoside RT inhibitor therapy. *J. Antimicrob. Chemother.*, 59(5):1013–1016.

Pao, D., Andrady, U., Clarke, J., Dean, G., Drake, S., Fisher, M., Green, T., Kumar, S., Murphy, M., Tang, A., Taylor, S., White, D., Underhill, G., Pillay, D., and Cane, P. (2004). Long-term persistence of primary genotypic resistance after HIV-1 seroconversion. *J. Acquir. Immune Defic. Syndr.*, 37(5):1570–1573.

Romano, L., Venturi, G., Bloor, S., Harrigan, R., Larder, B. A., Major, J. C., and Zazzi, M. (2002). Broad nucleoside-analogue resistance implications for human immunodeficiency virus type 1 reverse-transcriptase mutations at codons 44 and 118. *J. Infect. Dis.*, 185(7):898–904.

Suñé, C., Brennan, L., Stover, D. R., and Klimkait, T. (2004). Effect of polymorphisms on the replicative capacity of protease inhibitor-resistant HIV-1 variants under drug pressure. *Clin. Microbiol. Infect.*, 10(2):119–126.
